# Motor Cortical Plasticity to Training Started in Childhood: The Example of Piano Players

**DOI:** 10.1371/journal.pone.0157952

**Published:** 2016-06-23

**Authors:** Raffaella Chieffo, Laura Straffi, Alberto Inuggi, Javier J. Gonzalez-Rosa, Francesca Spagnolo, Elisabetta Coppi, Arturo Nuara, Elise Houdayer, Giancarlo Comi, Letizia Leocani

**Affiliations:** 1 Department of Neurology, Scientific Institute Hospital San Raffaele, Milan Italy; 2 Experimental Neurophysiology Unit, Institute of Experimental Neurology (INSPE), Scientific Institute Hospital San Raffaele, Milan Italy; 3 University Vita-Salute San Raffaele, Milan, Italy; Universita degli Studi di Verona, ITALY

## Abstract

Converging evidence suggest that motor training is associated with early and late changes of the cortical motor system. Transcranial magnetic stimulation (TMS) offers the possibility to study plastic rearrangements of the motor system in physiological and pathological conditions. We used TMS to characterize long-term changes in upper limb motor cortical representation and interhemispheric inhibition associated with bimanual skill training in pianists who started playing in an early age. Ipsilateral silent period (iSP) and cortical TMS mapping of hand muscles were obtained from 30 strictly right-handed subjects (16 pianists, 14 naïve controls), together with electromyographic recording of mirror movements (MMs) to voluntary hand movements. In controls, motor cortical representation of hand muscles was larger on the dominant (DH) than on the non-dominant hemisphere (NDH). On the contrary, pianists showed symmetric cortical output maps, being their DH less represented than in controls. In naïve subjects, the iSP was smaller on the right vs left abductor pollicis brevis (APB) indicating a weaker inhibition from the NDH to the DH. In pianists, interhemispheric inhibition was more symmetric as their DH was better inhibited than in controls. Electromyographic MMs were observed only in naïve subjects (7/14) and only to voluntary movement of the non-dominant hand. Subjects with MM had a lower iSP area on the right APB compared with all the others. Our findings suggest a more symmetrical motor cortex organization in pianists, both in terms of muscle cortical representation and interhemispheric inhibition. Although we cannot disentangle training-related from preexisting conditions, it is possible that long-term bimanual practice may reshape motor cortical representation and rebalance interhemispheric interactions, which in naïve right-handed subjects would both tend to favour the dominant hemisphere.

## Introduction

Plasticity represents the ability of the central nervous system to react to physiological and pathological events, occurring during normal development, environmental stimuli (i.e. learning) and pathological processes[1]. For instance, learning new tasks has been shown to be associated with immediate and long lasting plastic changes in the sensorimotor cortex and in several associative areas in monkeys [2, 3] and in humans [4–6]. Motor training in piano players leads to bimanual hand dexterity. Pianists show, indeed, a more symmetric motor performance and left-hand superiority in motor tasks in comparison with right-handed naïve controls [7–9]. These behavioural differences have been associated with a functional reorganization of motor circuits and with structural brain changes as well. First, functional magnetic resonance imaging (fMRI) studies have demonstrated lower ipsilateral motor cortex activation during unilateral movement [10, 11] and reduced recruitment of motor association areas (such as premotor cortex, cerebellum, prefrontal or basal ganglia) during bimanual coordination [12] in piano players compared to naïve subjects. These data have been interpreted as the result of a long lasting change in the functional properties and connectivity in sensorimotor cortex after extensive hand training [13, 14]. Moreover, a reduced interhemispheric asymmetry of the intrasulcal length of the precentral gyrus [7, 15] and an increase in grey matter volume of the sensorimotor cortex [16] have been observed in musicians in comparison with naïve subjects. Transcranial magnetic stimulation (TMS) mapping allow to study plastic rearrangements of the cortical motor outputs. For instance, a larger representation of the dominant hemisphere in right-handers is the typical pattern of cortical motor mapping associated with handedness in healthy subjects [17–20]. Enlargement of motor cortical representation has been reported after 5 days of unilateral keyboard training, with a decrease after consolidation of the motor task [21]. Neurophysiological changes occurring in the motor representations after long-term bimanual skilled learning are, instead, still largely unknown.

Musicians show also greater capability of controlling hand independence than controls [9]. Mirror movements (MMs) refer to unintended movements on one body segment mirroring the contralateral voluntary movement [22, 23]. Although, MMs have been described in pathological conditions such as cerebral palsy, Parkinson disease and stroke [24–26], they are not necessarily a pathological sign. MMs can, indeed, be observed also in healthy subjects [27], mainly in relation with complex motor task or fatigue [28, 29] as well as in relation with handedness [30]. For instance, in right-handers, the occurrence of MMs is lower during movement of the dominant than the non-dominant hand [31] suggesting an asymmetric ability to control the homologous hemisphere during unilateral movements. Transcallosal motor inhibition is considered particularly important during strictly unilateral movements, in order to inhibit unintentional ipsilateral movements [32]. In physiological conditions the MMs occurrence has been mainly related to an incomplete transcallosal inhibition of one motor cortex from the contralateral [27]. A larger anterior corpus callosum size has been reported in pianists compared to controls [33] but whether motor practice may increase interhemispheric inhibition thus improving the MMs control has not yet been investigated. TMS is a useful technique also for studying functional transcallosal connectivity between the two motor cortices [20, 34]. The ipsilateral silent period (iSP) consists in stimulating one motor cortex during maximal voluntary contraction of the ipsilateral hand [35]. The stimulated motor cortex would induce a transcallosal interhemispheric inhibition of the opposite motor cortex [36]. This inhibition is detectable as a pause in the ipsilateral electromyographic (EMG) trace. ISP represents, therefore, a direct measure of the interhemispheric control of voluntary cortical motor output [37].

In the present study we used TMS to test the hypothesis whether, in pianists, hand dexterity as well as the capability of controlling hand independence would be related with more symmetrical motor cortex representation and symmetrical interhemispheric interaction compared with controls. Fine motor skill learning, such as music playing, can induce cerebral plastic changes that still need to be better understood. Fine motor skill practice, such as music playing, can induce functional and structural cerebral plastic changes that still need to be better understoodThe functional and structural brain differences between pianists and naive subjects have been mainly related to a different brain development in musicians, who often started playing the piano at an early age. For instance, in the first decade of age, during the development of the corpus callosum, bimanual training may induce plastic changes in callosal fibres before a complete myelinization takes place [33]. This could lead to long-term changes in transcallosal functional connections which could be revealed by interhemispheric inhibition measurements. Early-trained musicians show greater connectivity in the posterior midbody/isthmus of the corpus-callosum and MRI fractional anisotropy in this region seems to be related to age of onset of training. It has been proposed that training before the age of 7 years results in changes in white-matter connectivity [38]. Moreover, the amount of practice seems to have a relevant impact on brain structure, not only during the sensitive period of childhood but throughout life [9]. So far, professional pianists have been selected in the majority of studies performed on musicians [39, 40]. Full-time musicians often start playing piano at childhood and continue intensive practice as adults, making difficult to distinguish the relative contribution of the two factors for data interpretation. In order to preferentially evaluate the effect of early bimanual motor training on brain function, we selected subjects who started playing piano at an early age but who had discontinued intense practice at the time of study entry.

## Materials and Methods

### Participants

A total of sixteen pianists (7F; age 27.5 + 4.3 years) and 14 controls (7F; age 26.9 + 3.5 years), with no history of neurological illness, gave their written informed consent to participate in the study, which was approved by the Ethics Committee of the ‘Ospedale San Raffaele’ (protocol number: 58/2008). All subjects were fully right-handed, having obtained the maximal score at a translated modified version of the Edinburgh Handedness Inventory [41]. Pianists started to play piano before their first decade of life (mean age 7 ± 1.4 years). They practiced for at least six years for 10–21 hours per week (median 14 hours) and they continued as hobby at the time of study entry, playing a few hours during week-ends. Controls never played any musical instrument.

### Hand dexterity

Hand dexterity was assessed using:

- The Nine Hole Peg Test (NHPT) score [42] (16 pianists and 14 controls): consisting in the time taken by the subject to insert every peg in the empty holes and then remove them and place them back in the shallow container, as quickly as possible. Both the dominant and non-dominant hands were tested twice and the best time for each side was taken for subsequent analysis.
- Finger tapping (FT) (12 pianists and 12 controls): subjects were comfortably seated on a chair, their forearm and hands resting on a table placed in front of them. Participants were instructed to press on a left-button mouse as fast as possible during 10 s with their index finger. The test was performed three times, with both hands, in random sequence. Tapping frequency was calculated using STIM software (Compumedics GermanyGmbH, Singen, Germany). The mean frequency of the three trials, for each hand, was kept for analyses [20].

### Transcranial magnetic stimulation

Subjects were seated on a comfortable armchair in a quiet room, with their eyes open, elbows semiflexed and forearms pronated and supported by a pillow. A Magstim 200 stimulator (Magstim Comp.,UK) connected to a figure-of-eight coil (70 mm) was used. The coil was placed tangentially to the scalp with the handle oriented posteriorly. Motor evoked potentials (MEPs) from abductor pollicis brevis (APB), adductor digiti minimi (ADM), extensor carpi radialis (ECR) muscles of both sides were recorded simultaneously, at rest, using Ag/AgCl surface electrodes in a belly-tendon montage. Electromyogram (EMG) was recording using a SCAN 4.1 (Synamps Amplifiers, Neuroscan Inc., Herndon, VA), bandpass filtered at 30–1000 Hz. Impedances were kept below 5 kΏ. The amplified analog outputs were digitized at 2 kHz and stored on a personal computer for off-line analysis.

Among the included subjects, 12 pianists (7F; age 26.7 ± 3.7 years) and 12 controls (4F; age 26.8 ± 4.8 years) underwent TMS mapping. A grid with 1 cm squares was positioned on the vertex of a closely-fitting cap. The hemisphere to be stimulated first was chosen in each subject in random order. The cortical hotspot was defined as the grid node eliciting optimal MEPs on at least one of the contralateral APB or ADM muscles. For each side, resting motor threshold (RMT) was measured over the hotspot as the minimal intensity evoking MEPs in at least one of the two muscles with amplitude of 50 μV or higher in 5 out of 10 stimuli, using 5% increment or decrement, starting from 50% of max stimulator output [43]. The intensity used for mapping was set at 115% RMT. Stimuli were delivered every 4 s at each grid node starting from the hotspot and then successively stimulating adjacent sites until no MEPs were evoked. The whole procedure was repeated to have 4 stimulations for each active site. The level of background EMG activity was constantly monitored during the experiment.

The iSP, as suppression of the ongoing voluntary EMG activity in the APB muscle induced by focal TMS over the ipsilateral motor cortex [34], was assessed in 11 pianists (5F, age 26.4 ± 1.2 years) and 11 controls (5F, age 28.5 ± 4.3 years). Fifteen cortical stimuli at 90% of the maximal stimulator output were delivered on the ipsilateral hand motor cortex while subjects maintained a maximal APB tonic contraction.

### Mirror movements recording

**Fig 1:**
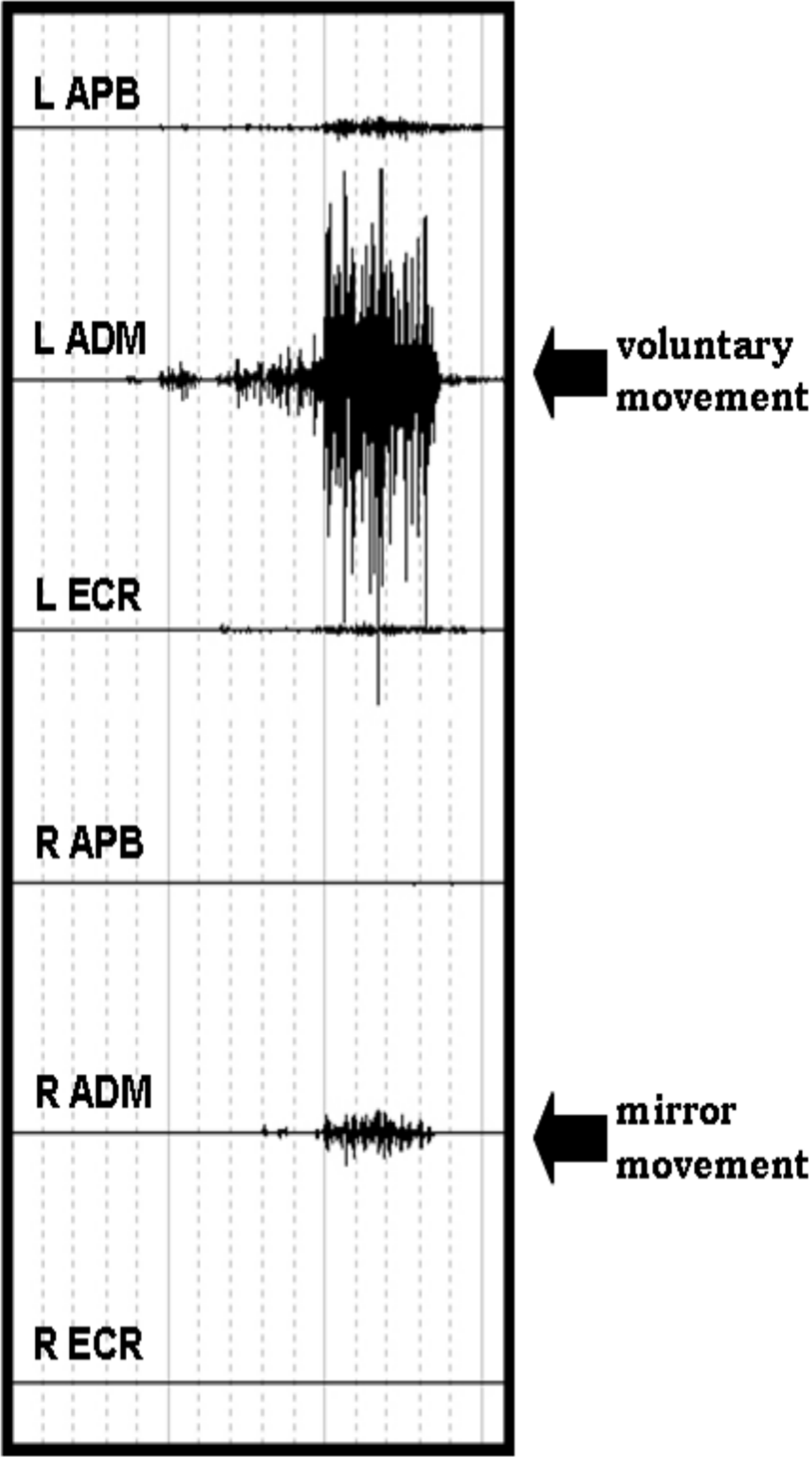
example of mirror movement in the right ADM during voluntary movement of the contralateral homologous muscle in a control subject.

Participants were comfortably seated on an armchair, their forearms and pronated hands resting on a table in front of them. All the subjects were asked to perform a single voluntary fast phasic contraction of APB, ADM and ECR muscles after a vocal “go signal”, in 3 separate sessions (one for each muscle). For each muscle, five trials were recorded with an interval of 5–6 seconds. Between trials, subjects were asked to stay completely at rest and EMG was constantly monitored checking for any muscle pre-contraction of both sides. For each muscle, MM score of 1 was assigned when EMG activity in the homologous muscle was visually detected on at least one of five trials. Patients performed the same task with both arms separately (Fig 1).

### Offline data analyses

**Fig 2:**
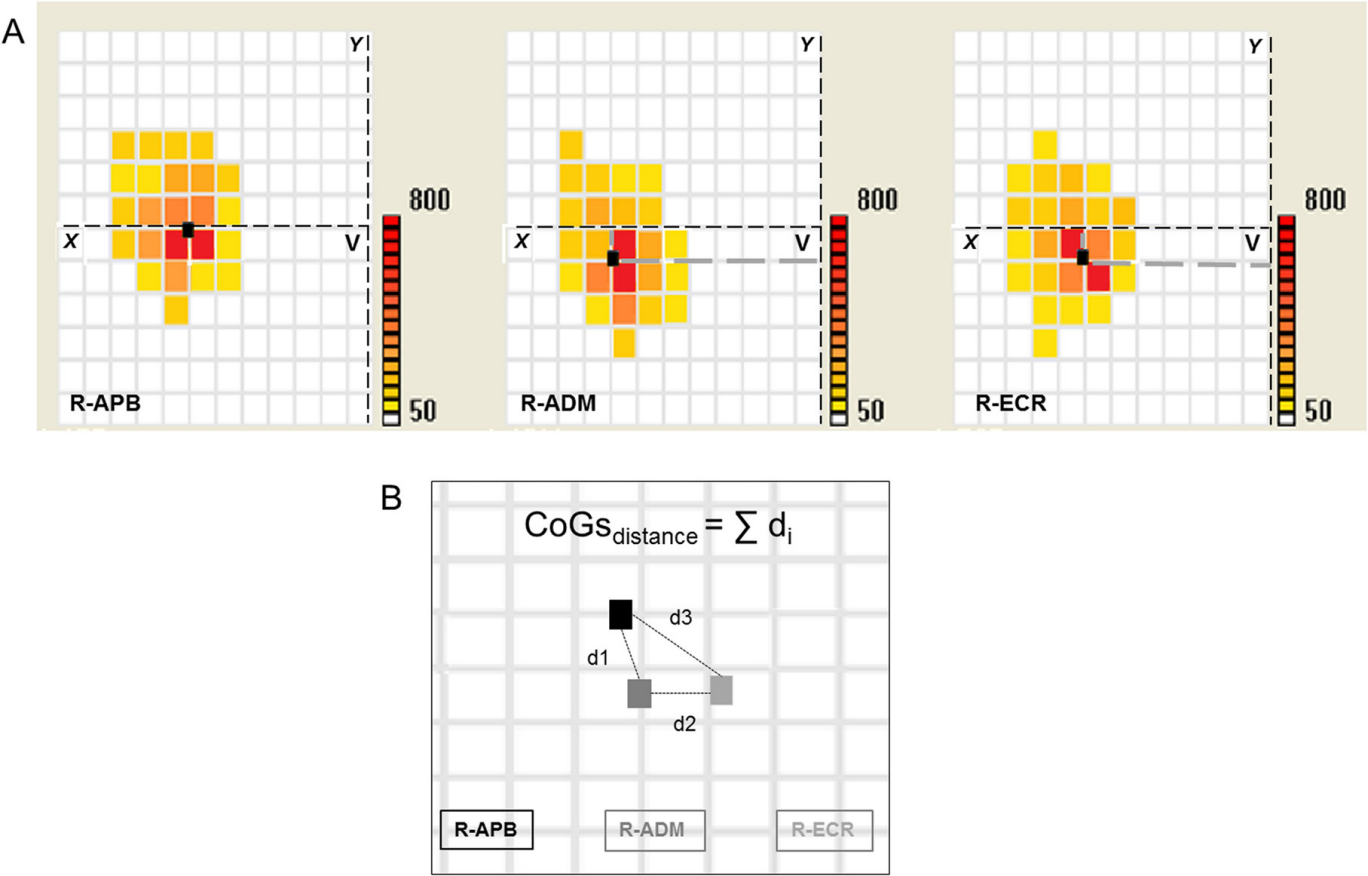
example of muscles CoGs calculations and their reciprocal distance. (A) CoG position: within each participant’s muscle representation, the CoG represents the average coordinate of all the responsive sites, weighted by their corresponding MEP intensity. (B) CoGs_distance_: distances (d_i_) between each CoG pairs were calculated and then summed.

Considering TMS mapping, the peak-to-peak amplitudes of four MEPs obtained from the stimulation of each scalp position were measured and averaged for each of the three muscles. The following map parameters were then calculated:

1. Maximal amplitude (μV) (peak-to-peak MEPs amplitude) in the hotspot, mean of three muscles studied.
2. Number of responsive sites (map_area_, in cm^2^) at which a MEP of amplitude over 50 μV was evoked, mean of three muscles studied.
3. Centre-of-gravity (CoG) as the average of stimulated position coordinates weighted by MEP intensity, calculated as following:
  $CoG_{x}=\frac{\left(\sum_{x}MEP_{x}*X\right)}{\sum_{x}MEP_{x}}$ $CoG_{y}=\frac{\left(\sum_{y}MEP_{y}*Y\right)}{\sum_{y}MEP_{y}}$ where MEP_X_/ MEP_Y_ are the intensities of the MEP value at X/Y-th grid position (Fig 2A). The proximity of hand muscle representations (ADM, APB, ECR) was measured by summing the relative distances of the CoGs (CoGs_distance_) of three muscles. The lower was the value the closer the motor maps of the three muscles (Fig 2B).
4. Overlap of three motor maps was estimated as the ratio between the average of the three separate areas and the whole area of three muscles superimposed, so a complete overlap corresponded to the value of 1 while a complete maps’ segregation to the value of 0.

The iSP was quantified in the average of the fifteen single rectified-EMG traces. The iSP_onset_ latency was defined as the time point, after TMS pulse, with EMG activity constantly smaller (for at least 10 msec) than the averaged baseline EMG contraction (pre-stimulus between -50 and -20 msec). The iSP_offset_ was defined as the first time point after iSP_onset_ in which the level of EMG activity regained the baseline value. The iSP_duration_ was measured as the difference between iSP_offset_ and iSP_onset_. The iSP_area_ (in mV*s), was calculated as following [44]:

$$iSP_{area}=iSP_{amplitude}*iSP_{duration}.$$

In order to reduce inter-subject variability related to the degree of pre-stimulus contraction, iSP_area_ was normalized for the rectified baseline EMG activity between -50 msec and -20 msec pre stimulus as:

$$n-iSP_{area}=\left[\frac{\left(EMG_{area}-iSP_{area}\right)}{EMG_{area}}\right]*100$$

The asymmetry index (AI) for map_area_ n-iSP_area_, iSP_duration_ and NHPT was calculated as:

$$AI=\frac{\left(\text{dominant - not dominant}\right)}{\left(\text{dominant}\;+\;\text{not dominant}\right)}$$

In the AI, ranging from 1.0 to -1.0, positive values indicate larger measurements obtained on the dominant side, while 0 indicates a perfect symmetry.

### Statistical analysis

Data were analysed with SPSS 13.0 (Technologies, Inc. Chicago, USA). After verifying the normal distribution by Kolmogorov-Smirnov test, parametric or non-parametric statistical tests were used. According to data normality, a mixed factorial ANOVA designed for repeated measurements was used for the neurophysiological variables (RMT, MEPs amplitude, map_area_, CoGs_distance_, overlap, background EMG iSP_duration_ and n-iSP_area_) and the Conover’s free distribution method, a non-parametric ANOVA based on ranks, for the hand dexterity measures (NHPT and FT) [45]. In both cases, “side” (two levels: dominant and non-dominant) was considered as a within-subjects factor and “group” (two levels: pianists and controls) as a between-subjects factor. In a main effect was found, post-hoc analyses were performed using independent t-test or the equivalent non parametric test (Mann-Whitney test) and paired t-tests or the equivalent non parametric test (Wilcoxon Signed Rank test) for the between and within group comparisons, respectively. Differences in the asymmetry index between pianists and controls were evaluated using independent Student’s t-tests for NHPT, FT and map_area_ and Mann-Whitney test for n-iSP_area_ and iSP_duration_. The number of subjects showing MMs (at least one of the 3 recorded muscles) in pianists and controls was compared using Chi square (Fisher's exact test). For each group differences in MMs occurrence between the left and right side were explored using the McNemar's test. We also considered differences in n-iSP_area,_ AI-NHPT, AI-FT, AI-map_area_-AI iSP_duration_ and AI-iSP_area_ according to the occurrence or not of MMs subjects using Mann-Whitney test.

Spearmann's correlation coefficient was used to test the relationship among the asymmetry indices of neurophysiological parameters (map_area,_ iSP_duration_ and n-iSP_area_) and hand dexterity (NHPT and FT). Significance level was set at p ≤0.01 after multiple comparison correction.

## Results

### Hand dexterity

**Fig 3:**
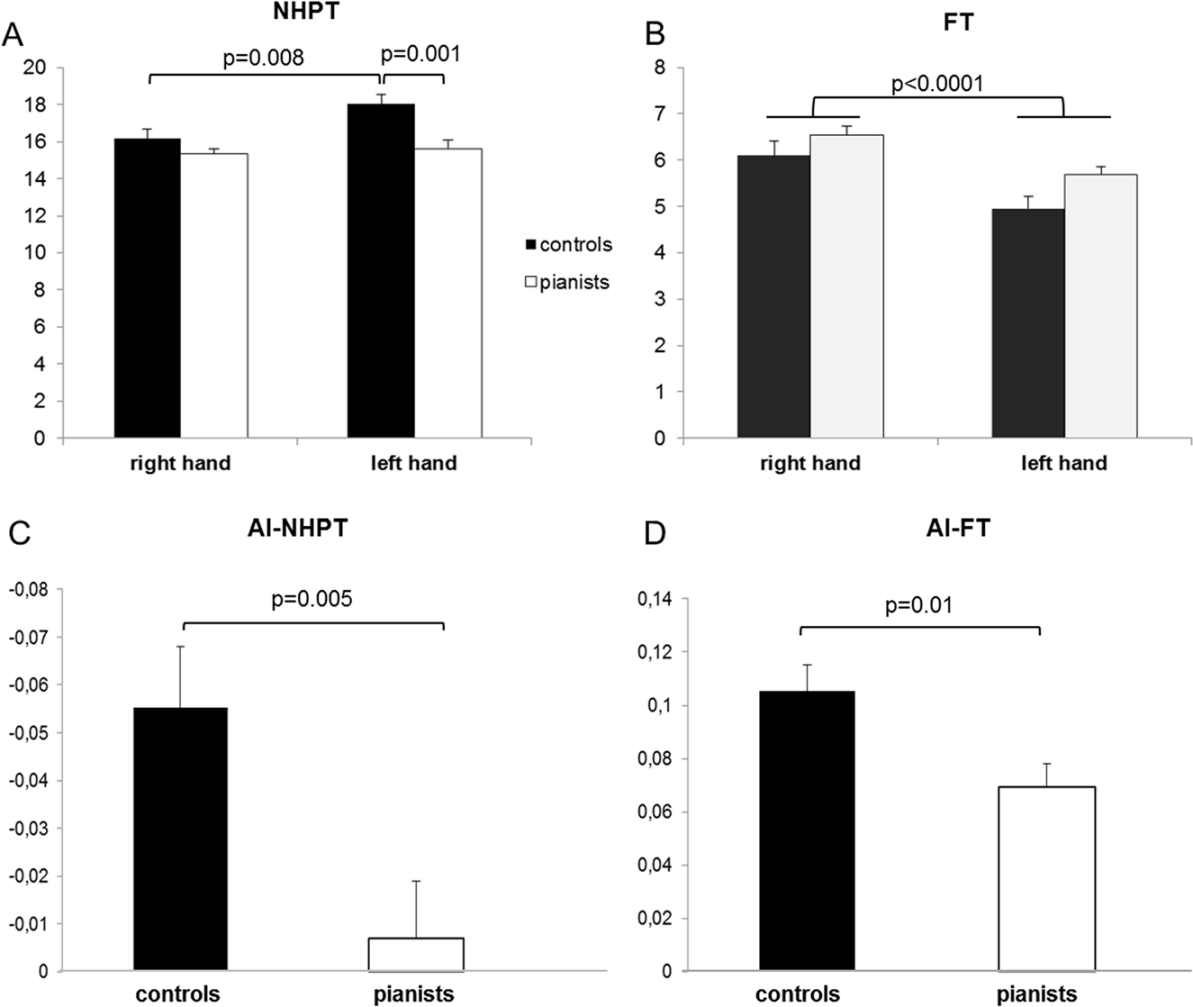
Behavioral results. A. Nine hole peg test (NHPT) score: left hand in pianists was significantly faster than in controls (p = 0.001). In the control group the time required to perform NHPT was shorter for the right than the left hand (p = 0.003). B Finger tapping (FT) scores: a significant effect of “side” factor was observed (F_1,22_ = 100.6, p<0.0001) being the right hand faster than the left hand. A trend was observed for the “group” comparison (F_1,22_ = 3.8, p = 0.06) and the “side” x “group” interaction was not significant (F_1,22_ = 0.18, p = 0.6). C. NHPT asymmetry index (AI) score was significantly lower in pianist than in controls indicating more symmetric motor performance in pianists (p = 0.005) D. FT AI was significantly lower in pianists than in controls (p = 0.01).

The ANOVA analysis performed for NHPT showed a significant effect of “side” (F_1,28_ = 25, p<0.0001) and “group” (F_1,28_ = 9.5, p = 0.005) factors; “side” x “group” interaction was also statistically significant (F_1,28_ = 6.32, p = 0.018). For the dominant hand NHPT score was similar in the two groups (p = 0.3), while the left hand was faster in pianist than in controls (p = 0.001). In the control group the time required to perform NHPT was shorter for the right than the left hand (p = 0.003). In pianists the two hands executed the motor task approximately in a similar time (p = 0.2) (Fig 3A, Table 1, S1 Database). Asymmetry index between the two groups was significantly different, being lower for pianist (p = 0.005) (Fig 3C, Table 2, S1 Database).

**Table 1:** Pianists and controls behavioural and neurophysiological data. MEP_amplitude_ and MAP_area_ are reported as average of APB, ADM and ECR muscles. We included in the table t (or Z) and p values of the post-hoc comparisons if a significant interaction between “group” and “side” factors was obtained from the ANOVA analysis, as well as F and p values if a significant effect of “group” or/and “side” factors was obtained from the ANOVA analyses without significant interaction between factors.

					statistics	
	Side	Controls	Pianists	DH vs NDH	DH vs NDH	*Controls vs pianists*
				Controls	Pianists	
	**DH**	16.2±1.6	15.5 ±0.9			n.s.
**NHPT**				Z = 2.9; p = 0.003	n.s.	
**(sec)**	**NDH**	18.2±1.8	15.9±1.8			U = 36.5; Z = 3.14; p = 0.001
	**DH**	6.1±1.1	6.5±0.7			
**FT**				F_1,22_ = 100.6; p<0.001	F_1,22_ = 100.6; p<0.001	F_1,22_ = 3.8; p = 0.06
	**NDH**	4.9±0.9	5.6±0.6			
	**DH**	46±5	46±6			
**RMT**				n.s.	n.s.	n.s.
**(%)**	**NDH**	48±6	46±5			
	**DH**	638±417	710±447			
**MEP**_**amplitude**_				n.s.	n.s.	n.s.
_**(**_μ**V)**	**NDH**	591±279	546±278			
	**DH**	18.3±7.1	12.9±3.4			t_(22)_ = 2.4; p = 0.02
**Map**_**area**_				t_(11)_ = 4.45; p = 0.001	n.s.	
**(cm**^**2**^**)**	**NDH**	12.1±3.7	13.8±4.3			n.s.
	**DH**	0.86±0.1	0.85±0.1			
**CoG**_**distance**_				F_1,22_ = 15; p = 0.001	F_1,22_ = 15; p = 0.001	n.s.
**(cm)**	**NDH**	1.58±0.2	1.52±0.3			
	**DH**	0.8±0.09	0.6±0.07			t_(22)_ = 5.1; p<0.001
**Overlap**				t_(11)_ = 6.7; p<0.001	n.s.	
	**NDH**	0.6±0.09	0.5±0.09			t_(22)_ = 2; p = 0.06
	**DH**	224,5±128,3	201,8±109,5			
**EMG**				n.s.	n.s.	n.s.
	**NDH**	245,5±142,4	211,9±102,7			
	**DH**	50.1±3.3	56.9±2.7			n.s.
**n-iSP**_**area**_				t_(10)_ = 3.7; p = 0.004	n.s.	
	**NDH**	37.9±3.9	53.9±3.1			t_(20)_ = 3.2; p = 0.004
	**DH**	44±8	41.5±10			
**iSP**_**duration**_				n.s.	n.s.	n.s.
**(msec)**	**NDH**	42.0±13	38.8±12			

Abbreviations: DH/NDH = dominant/non dominant hemisphere or hand; NHPT = nine hole peg test; RMT = resting motor threshold; MEP = motor evoked potentials; n-iSP_area =_ normalized iSP_area_; CoG = center of gravity; n.s. = not significative

**Table 2:** Asymmetry index (AI) for map_area_ n-iSP_area_, iSP_duration_ and NHPT in pianists and controls. The AI range from 1.0 to -1.0 and the value of 0 corresponds to perfect symmetry between the two sides (Dominant = Non Dominant).

	AI-NHPT	AI-FT	AI-map_area_	AI-iSP_duration_	AI-iSP_area_
	(14 C *vs* 16 P)	(12 C *vs* 12 P)	(12 C *vs* 12 P)	(11 C vs 11 P)	(11 C *vs* 11 P)
**Controls (C)**	-0.06±0.04	0,11±0,03	0.18±0.13	-0.04±0.19	-0.15±0.15
**Pianists (P)**	-0.01±0.04	0,07±0,03	-0.03±0.15	-0.04±0.17	-0.03±0.09
**statistics**	t_(28) =_ -3.1	t_(22) =_ 2.7	t_(22)_ = 2.8	n.s.	t_(20)_ = 2.2
	= 0.005	p = 0.01	p = 0.009		p = 0.04

Abbreviations: NHPT = nine hole peg test; FT = finger tapping; n-iSP_area =_ normalized iSP_area_; n.s. = not significative

Considering the FT task, a significant “side” effect was observed (F_1,22_ = 100.6, p<0.0001) being the right hand faster than the left hand (Fig 3B). A trend was observed for the “group” comparison (F_1,22_ = 3.8, p = 0.06) and the “side” x “group” interaction was not significant (F_1,22_ = 0.18, p = 0.6) (Fig 3B). The FT-AI resulted significantly lower in pianists compared to controls (p = 0.01) (Fig 3D, Table 2, S1 Database)

### Focal TMS mapping

The ANOVA analysis did not indicate any significant effect of both “side” and “group” on RMT and on MEPs amplitudes (average and standard deviation in Table 1, S2 Database).

**Fig 4:**
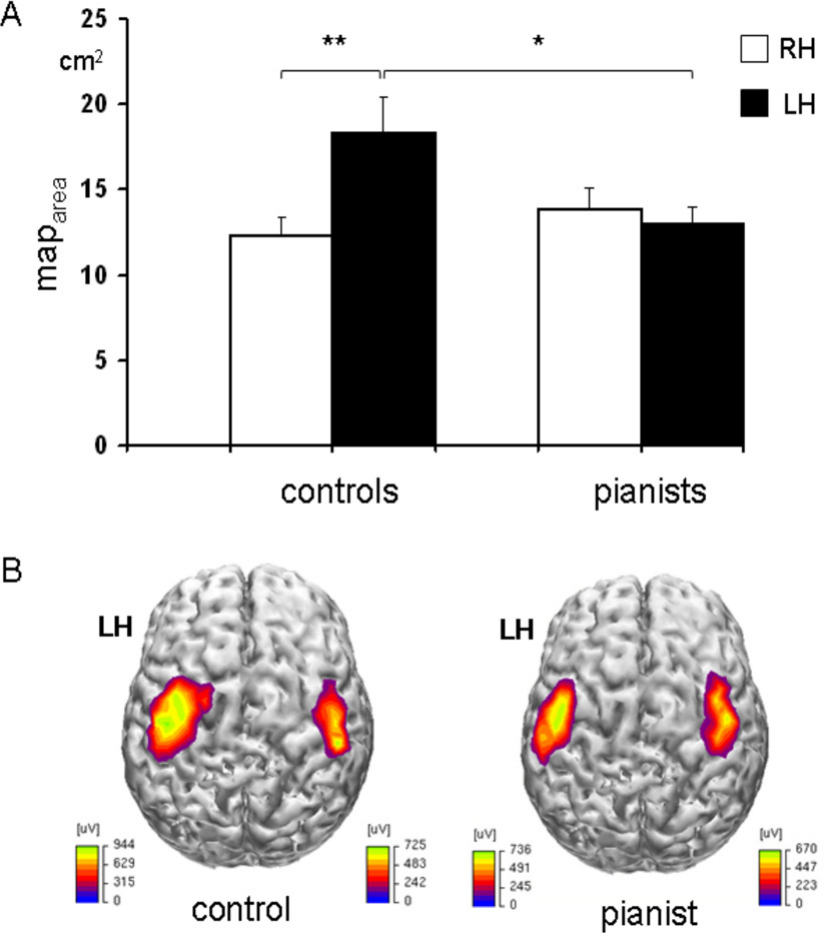
Cortical motor representation of the hand muscles (mean of APB, ADM and ECR) over the dominant (LH) and non-dominant (RH) hemisphere in pianists and controls (mean and standard error). **A.** Map_area_ in the dominant hemisphere of the control group was significantly larger compared with their non-dominant hemisphere (** p = 0.001) and with the dominant hemisphere of pianists (*p = 0.029). **B**. Example of cortical motor mapping of ADM in a pianist and a control naïve subject. MEPs amplitudes higher than 50 mV were interpolated and projected on an average brain cortical surface reconstruction using Curry software V4.6. The interhemispheric asymmetry in map_area_, with larger representation of the dominant hemisphere, is of note only in the naïve subject.

For map_area_ a significant effect of “side” (F_1,22_ = 8.8 p = 0.007), and “side” x “group” interaction (F_1,22_ = 16.241; p = 0.001) was found, while “group” was not significant (F_1,22_ = 1.1 p = 0.3). The post-hoc analysis revealed that map_area_ over the dominant hemisphere was smaller in piano players than in controls (p = 0.029), while no difference was observed for the non-dominant hemisphere (p = 0.3). Map_area_ was significantly larger on the dominant than non-dominant side in controls (p = 0.001), while did not significantly differ in piano players (p = 0.4) (Fig 4, S2 Database). Map_area_ asymmetry index significantly differed between the two groups (p = 0.009) being the degree of asymmetry between the left and right hemispheres greater in controls than in pianists (Table 2, S2 Database). Considering the CoGs_distance_ a significant effect of “side” (F_1,22_ = 15; p = 0.001) was found being muscles CoGs closer over the dominant than the non-dominant hemisphere and no significant effect of “group” (F_1,22_ = 0.05; p = 0.8) and interaction between the two factors (F_1,22_ = 0.02; p = 0.8) were found. Regarding the overlap measure, a significant effect of “side” (F_1,22_ = 34; p<0.001) and “group” (F_1,22_ = 19; p<0.001) factor was observed. There was also a significant interaction between the main factors (F_1,22_ = 11; p = 0.003). The dominant hemisphere in controls showed a greater overlap of maps of the 3 muscles (APB, ADM, ECR) compared to their non-dominant hemisphere (p<0.001) and to the dominant hemisphere of pianists (p<0.001) (Table 1, S2 Database).

### Ipsilateral silent period

We first checked for any differences in maximal APB tonic contraction which could possibly influence the iSP measurements. No “side” (F_1,19_ = 2.1, p = 0.16), “group” (F_1,19_ = 0.4, p = 0.49), or “side” x”group” (F_1,19_ = 0.6, p = 0.42) effects on background EMG were obtained from the ANOVA analysis (average and standard deviation in Table 1, S3 Database).

**Fig 5:**
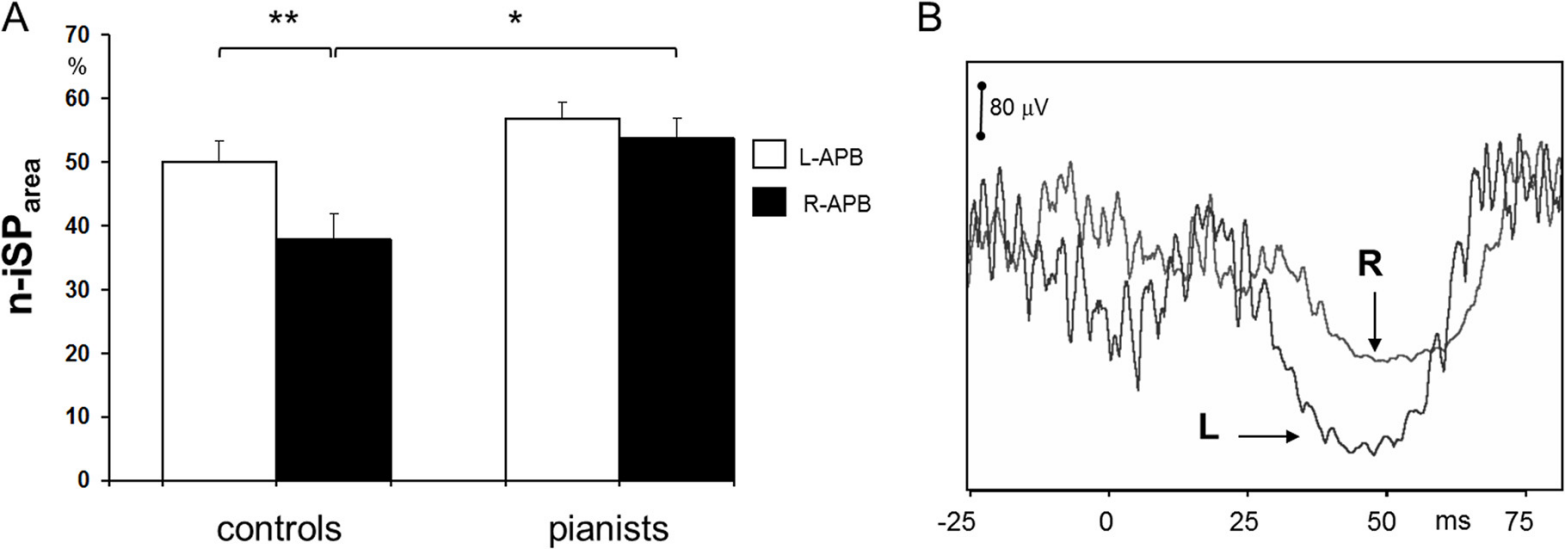
**A.** Average normalized ipsilateral silent period area (n-iSP_area_) on the left and right APB in pianists and naïve controls, the latter showing a wake suppression of voluntary EMG on the right APB to stimulation of the non-dominant ipsilateral hemisphere (error bars: standard error of the mean. Left *vs* right APB in controls **p<0.001; controls *vs* pianists R-APB *p = 0.008). **B.** Example of ipsilateral silent period on right APB (R) and left APB (L) in a control naïve subject. Note the stronger inhibition of voluntary EMG on the left, non-dominant side by stimulating the ipsilateral left, dominant hemisphere.

The ANOVA analysis performed for the n-iSP_area_ revealed a significant effect of both “side” (F_1,19_ = 14.8 p = 0.002) and “group” (F_1,19_ = 5.58 p = 0.01) as well as their interaction (F_1,19_ = 4.6 p = 0.04). The n-iSP_area_ was greater to stimulation of the dominant than the non-dominant hemisphere in the control group (p<0.001) whereas inhibition between the two hemispheres did not differ in piano players (p = 0.3). n-iSP_area_ to stimulation of the non-dominant hemisphere was larger in pianists than in controls (p = 0.004), no significant difference for dominant hemisphere between the two groups were observed (p = 0.13) (Fig 5, S3 Database). iSP_duration_ did not significantly differed between groups and hemispheres. n-iSP_area_ asymmetry index was significantly lower in pianist than in controls (p = 0.04) while iSP_duration_ asymmetry index was not significantly different (p = 0.9) (Table 2, S3 Database).

### Mirror Movements

MMs never occurred in piano players to movement of either hand (n = 0/16 subjects). In controls, MMs were never observed in relation with voluntary movement of the right muscles. Regarding voluntary movements of the left hand, no MMs were recorded on the ECR muscle. Conversely, five subjects showed MMs in relation with movements of the left ADM and two subjects showed MMs to movement of both APB and ADM. MMs occurred, therefore, only in control group (n = 7/14 subjects) during voluntary movement of the left hand. The number of subjects showing MMs in the control group was significantly higher than in pianists (p = 0.001-Fisher's exact test). In controls, MMs frequency to movement of the left hand was significantly higher compared to movements of their right hand (7/14 vs 0/14; p = 0.0233-McNemar's test).

In the five controls showing MMs on the right hand who underwent iSP assessment, the interhemispheric inhibition from the non-dominant to the dominant hemisphere was lower than in all the other subjects (U = 7, Z = 2.4, p = 0.01). Moreover, in subjects showing MMs on the right hand a greater inter-side asymmetry in favour of the dominant side was observed considering AI-NHPT (p = 0.005), AI- iSP_area_ (p = 0.002) and a trend for AI-map_area_ (p = 0.056) (Table 3).

**Table 3:** Asymmetry index (AI) according to Mirror movements (MMs) occurrence. The AI range from 1.0 to -1.0 and the value of 0 corresponds to perfect symmetry between the two sides (Dominant = Non Dominant).

	AI-NHPT	AI-FT	AI-map_area_	AI-iSP_duration_	AI-iSP_area_
	(7 *vs* 23)	(6 *vs* 18)	(6 *vs* 18)	(5 *vs* 17)	(5 *vs* 17)
**MMs**	-0.08 ±0.02	0.09±0.03	0.2±0.11	-0.08±0.27	-0.14±0.05
**no-MMs**	-0.02±0.22	0.08±0.03	0.03±0.17	-0.03±0.14	-0.07±0.14
**statistics**	U = 18; Z = -3.06	n.s.	U = 23; Z = -2.06	n.s.	U = 12; Z = -2.3
	p = 0.001		p = 0.04		p = 0.015

Abbreviations: NHPT = nine hole peg test; FT = finger tapping; n-iSP_area =_ normalized iSP_area_; n.s. = not significative.

### Correlations

**Fig 6:**
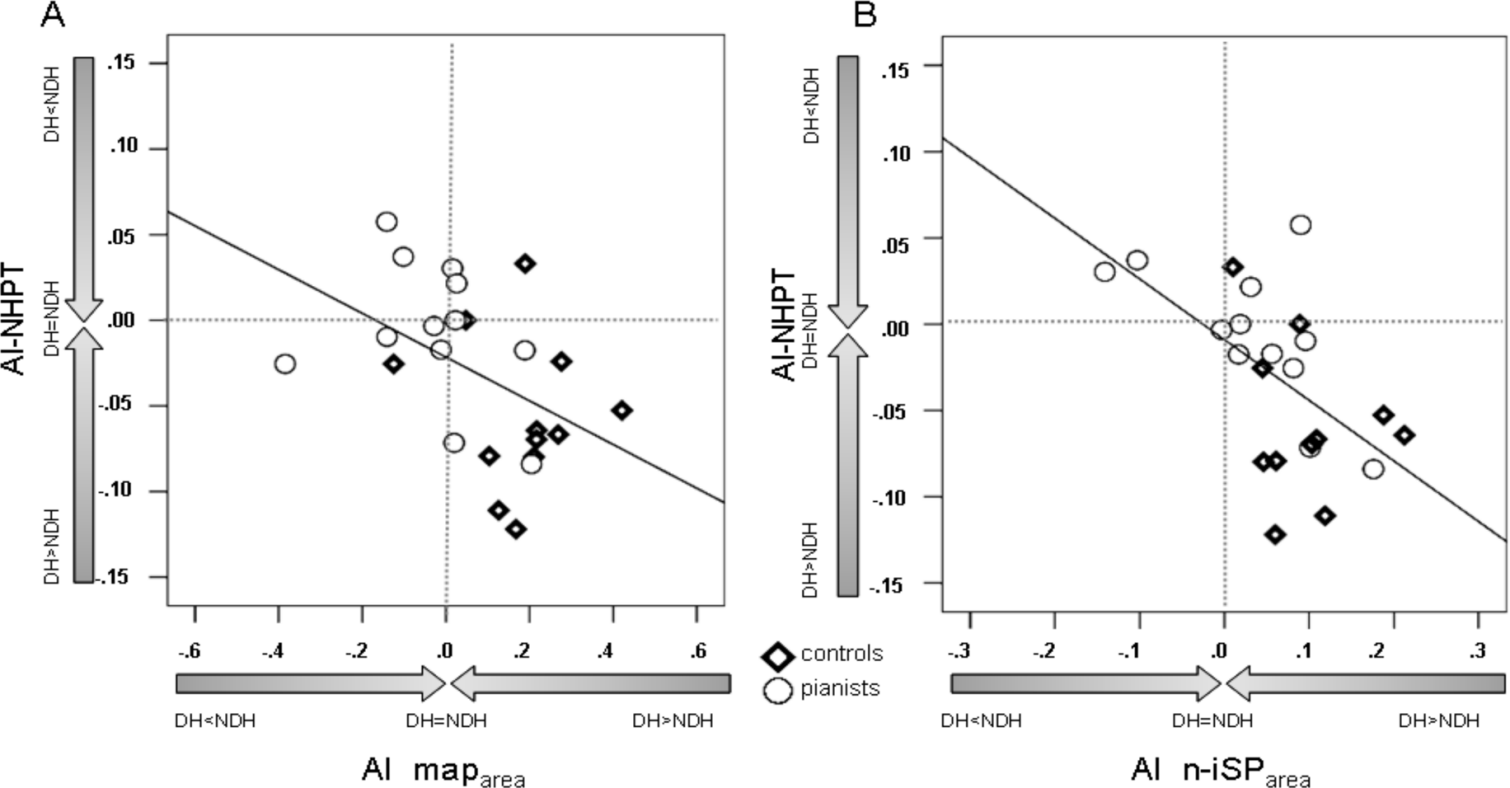
Correlation between the nine hole peg test (NHPT) asymmetry index (AI) and map _area_ AI and normalized-iSP_area_ (n-iSP_area_) AI. The degree of interhemipheric asymmetry in map_area_ (**A**) and n-iSP_area_ (**B**) correlate with the asymmetry in performing NHPT with the right and left upper limb (ρ = -0.48; p = 0.019 and ρ = -0.57; p = 0.004 respectively). Interhemispheric imbalance in both cortical representation and interhemispheric inhibition, favouring the dominant hemisphere, corresponds to a relatively slower non-dominant hand. Abbreviations: DH = dominant hemisphere or hand, NDH = non dominant hemisphere or hand.

Correlation analysis showed a trend for an inverse correlation between map_area_ asymmetry index and NHPT asymmetry index (ρ = -0.44; p = 0.032) (Fig 6A, Table 4). Therefore, a greater dominant map_area_ tended to correspond to a slower non dominant hand. Furthermore we found a significant inversely correlation between n-iSP_area_ asymmetry index and NHPT asymmetry index (ρ = -0.56; p = 0.007) (Fig 6B, Table 4). A greater inhibition of the dominant over non dominant hemisphere corresponds to a slower non dominant hand. No correlations were obtained between AI-NHPT and iSP_duration_. AI-FT do not correlated with any of the variable tested (AI-map_area_, AI- iSP_duration_ and AI-n-iSP_area_) (Table 4).

**Table 4:** correlation analysis among the asymmetry indices of neurophysiological parameters (map_area,_ iSP_duration_ and n-iSP_area_) and hand dexterity (NHPT and FT).

	AI-map_area_	AI-iSP_duration_	AI-iSP_area_
**AI-NHPT**	**ρ = -0.44; p = 0.032**	n.s.	**ρ = -0.56; p = 0.007**
**AI-FT**	n.s.	n.s.	n.s.

Significance level was set at p ≤0.01 after multiple comparison correction.

N.S. = not significant with p>0.05.

## Discussion

Our study demonstrated that piano players have a reduced degree of hand skill asymmetry as shown by FT performance in comparison with controls [8, 9]. However, performance of both left and right side was not significantly better in musicians than in controls. Considering that our group of pianists were playing just a few hours a week at time of study entry, these findings are in line with a previous work comparing FT performance in musicians with different practice regimes. The authors found a higher tapping scores in intensively practicing musicians, whereas less intensively practicing players were more similar to controls [9]. On the contrary, NHPT score showed better sensitivity in detecting differences in hand dexterity between groups. Greater inter-side symmetry of NHPT in pianists was mainly due to a lower score of their left hand (indicative of faster performance). NHPT has been used in previous studies on healthy subjects for detecting age-related changes in motor performance [20, 46]. Although previous studies on piano players assessed motor performance using single or sequential finger-tapping tasks, our data suggest that NHPT is a useful task, easy to perform, for evaluating changes in hand dexterity related to musical training.

Regarding TMS mapping, the finding of a larger motor cortical representation of the dominant hand muscles in naïve right-handed subjects is consistent with previous reports [17–20]. One of the main novel findings of the present study is that piano players showed a more symmetric hand motor representation, the dominant map area being reduced compared to naïve subjects. The reduction of motor cortical representation over the dominant hemisphere in pianists was associated with a lower degree of muscle map overlap than in controls, without a corresponding difference in the CoG_distance_. Thus, plasticity in piano players may be associated with a concentric reduction of fringes of excitability in the dominant motor maps, with higher segregation of single muscle representations. This plastic rearrangement of the relative representation of hand muscles in pianists could promote a rapid switch from co-activation to selective muscle activation and vice versa. Whether these findings are related to plasticity of local inhibitory circuits remains to be tested. Intracortical inhibition studies on expert piano players led to contrasting findings, mainly depending on the population and on the stimulation protocol [40]. In our study, intra-cortical inhibition and intra-cortical facilitation were not tested. We cannot, therefore, discuss their potential role in M1 plastic reorganization in pianists. Moreover, we can hypothesize that the reorganization of the motor output maps in pianists could be related not only to changes in local inhibitory circuits but also to the rearrangement of interhemispheric connections due to the acquisition of bimanual skills.n the present study controls displayed a motor cortical asymmetry favouring the dominant hemisphere not only consisting in larger motor maps but also in a stronger interhemispheric inhibition towards the non-dominant hemisphere. Both these measures were more symmetric in piano players. The efficacy of intherhemispheric inhibitory pathways was associated with behavioural effects, in particular concerning the occurrence of MMs. MMs are common and physiological in childhood due to immaturity of the motor system [27, 47]. In contrast, normal adults are usually able to perform strictly unilateral movements in daily life [22], although a slight, involuntary mirroring can often be detected with EMG even during relatively simple unimanual tasks [48]. A number of neurophysiological data suggest that ‘physiological’ mirroring depends on the activation of the crossed corticospinal tract originating from the M1 ipsilateral to the voluntary movement (mirror M1) mainly related to an incomplete transcallosal inhibition from one motor cortex to the other [48, 49]. In healthy right handers asymmetrical mirroring has been reported, with stronger MMs during voluntary movement of the non-dominant hand [31]. Consistently, in our sample, MMs appeared in naïve subjects during movements of the non-dominant hand. No MMs were recorded in pianists, indicating a more efficient control of strictly unilateral hand movement even if performed with the non-dominant side. A reduced occurrence of MMs can reflect a greater ability to inhibit the homolateral motor cortex during a simple unimanual motor task [48]. According to this hypothesis, pianists showed greater interhemispheric inhibition from the non-dominant to the dominant motor cortex. Furthermore, subjects with MMs had also a weaker interhemispheric inhibition from the non-dominant to the dominant hemisphere, as revealed by our iSP measures. Finally, our analyses indicated that asymmetric interhemispheric inhibition (as from iSP_area_) was correlated to asymmetric hand dexterity (as from NHPT), both favoring the dominant side. Our data of increased interhemispheric inhibition from the non-dominant to the dominant hemisphere in piano players are in apparent contrast with the finding of a reduced transcallosal inhibition in professional pianists using bi-hemispheric paired TMS [39]. However, in the latter study, data from the stimulation of left and right hemispheres were considered together, masking any possible differences between the dominant and non-dominant sides. Differences in the methodology between studies could also be responsible for different results, such as iSP vs bi-hemispheric TMS stimulation or the studied muscle (FDI vs APB). Moreover, several characteristics in the different populations, such as starting age and amount of daily practice, may have affected long-term cortical plasticity [9, 38] and thus explain contrasting findings among these studies.

With this study, we provide evidence for an improved interhemispheric communication, mainly due to inhibitory interactions, from the non-dominant to the dominant motor cortex in piano players. A possible explanation is that piano training would tend to balance interhemispheric inhibition, reducing the supremacy of the dominant over the non-dominant hemisphere. Asymmetric interhemispheric inhibition, advantaging the dominant over the non-dominant hemisphere would favor the dominant hand in approaching unimanual movements. Nevertheless, most bimanual movements performed in piano players require a similar level of skill between the two hands. Interestingly, a larger anterior corpus callosum in piano players who began musical training before the age of seven has been found compared with controls [33]. From a fractional anisotropy RMI study, it has been hypothesized that musical training can induce white matter plasticity if it occurs in a period when the involved fiber tracts are still under maturation. The authors proposed that increased myelination, caused by neural activity in fiber tracts during training, is one possible mechanism underlying the observed FA increases in the pyramidal tract in childhood practicing musicians [50].

Moreover, pianists show a lesser interhemispheric asymmetry in the intrasulcal length of the precentral gyrus (ILPG) in comparison with right-handed nonmusicians controls [7, 15].The ILPG is a measure of the motor cortex size and it also a correlate of the cortical motor hand representation evaluated with functional MRI and positron emission tomography (PET) techniques [51, 52]. Decreased anatomical asymmetry has been associated with a reduced asymmetry in distal hand/finger motor skills as assessed by index finger-tapping rates mainly caused by a more pronounced proficiency of the left, nondominant hand [7]. Therefore, the acquisition of bimanual motor skills, such as playing piano, produces in right handed subjects a more symmetric motor performance mainly related to an increase in left hand motor efficacy. This behavioural effect is associated with both structural and functional plastic changes of the motor system (i.e primary motor cortex and transcallosal connections).

However, it has also been hypothesized that structural brain differences in pianists would be due to inborn features and not to an effect of motor training [53]. Further studies are thus needed to assess the impact of preexisting factors, including genetics, that could influence brain changes associated with short and long-term motor learning. Indeed, we cannot exclude that the differences observed in our study between piano players and controls can be due to preexisting factors prompting or enhancing propensity to piano training.

## Conclusion

Piano players showed balanced motor cortical representations and interhemispheric inhibitory interactions, both of which tending to favor the dominant hemisphere in naïve subjects. These changes, consistent with enhanced local and interhemispheric cortical selectivity, may explain the reduction of involuntary mirroring with respect to naïve subjects (as found in the present study) and of cortical activation as observed in functional neuroimaging studies. Further exploration is needed to disentangle the time course of reshaping of cortical motor representation and interhemispheric interactions. Both phenomena may well occur in parallel, thus being reciprocal and interdependent, as it may be suggested by their correlation found in the present study.

## Supporting Information

S1 Database database of behavioural data. Abbreviations: C: controls, P: pianists, L: left, R: right, NHPT: nine hole peg test, FT: finger tapping, AI: asymmetry index.
(XLS)

S2 Database database of TMS mapping parameters. Abbreviations: C: controls, P: pianists, L: left, R: right, RMT: resting motor threshold, AI: asymmetry index.
(XLS)

S3 Database database of iSP (ipsilateral silent period) measurements. Abbreviations: C: controls, P: pianists, L: left, R: right, niSP: normalized ipsilateral silent period, AI: asymmetry index.
(XLS)

## Abbreviations

TMS: transcranial magnetic stimulation
iSP: Ipsilateral silent period
DH: dominant hemisphere or hand
NDH: than on the non-dominant hemisphere or hand
MMs: mirror movements
fMRI: functional magnetic resonance imaging
MEPs: Motor evoked potentials
APB: from abductor pollicis brevis
ADM: adductor digiti minimi
ECR: extensor carpi radialis
NHPT: nine hole peg test
EMG: electromyogram
RMT: resting motor threshold
CoG: centre-of-gravity
AI: asymmetry index
